# Febuxostat attenuates chronic kidney disease induced by adenine in rats: role of TLR4/NF-κB/NLRP3/Caspase1/IL-1β pathway

**DOI:** 10.1007/s10787-026-02308-0

**Published:** 2026-07-11

**Authors:** Omnia A. Nour, Mahmoud M. Samaha, Haitham M. Sewilam, Dalia H. El-Kashef

**Affiliations:** 1 Department of Pharmacology and Toxicology, Faculty of Pharmacy, Mansoura University, Mansoura, 35516 Egypt; 2 Department of Histology, Faculty of Medicine, Capital University (Formerly Helwan University), Cairo, 11795 Egypt; 3 Department of Histology and Cell Biology, General Medicine Practice Program, Batterjee Medical College, Jeddah, 21442 Saudi Arabia

**Keywords:** CKD, Adenine, Febuxostat, Oxidative stress, Inflammation

## Abstract

Chronic kidney disease (CKD) is a top public health priority and global health concern. Owing to the buildup of toxins and the decreased removal of inflammatory mediators, the illness frequently causes inflammation, which eventually impairs kidney function. This study sought to provide insight into the potential prophylactic effect of febuxostat against adenine-induced CKD. Rats were distributed into 4 groups; normal, adenine (200 mg/kg, orally, once daily) and febuxostat groups (rats received either 5 or 15 mg/kg of febuxostat concurrently with adenine). Febuxostat effectively attenuated adenine-induced CKD as manifested by marked drop in serum creatinine, BUN, proteinuria and rise in creatinine clearance. Histopathological analysis supported these biochemical results. Moreover, reduced MDA and increased GSH levels indicate that febuxostat restored the oxidant/antioxidant equilibrium. Additionally, febuxostat in both doses reduced inflammation as evidenced by reduced renal expression of TLR4 and NF-κB besides reduced renal levels of NLRP3, Caspase1, IL-1β and TNF-α. Collectively, febuxostat efficiently attenuated adenine-induced CKD via down-regulating TLR4/NF-κB/NLRP3/Caspase1/IL-1β signaling pathway. Thus, febuxostat could be beneficial in clinical use against CKD pending further analysis.

## Introduction

Chronic kidney disease (CKD) is rising in both developed and developing nations. This places an increasingly costly and demanding burden on health care systems that are already struggling with a lack of resources (Ali et al. 2019). CKD is a progressive disease which has no effective cure and could lead to major problems such diabetes, heart disease, and stroke, thus it necessitates complex and often costly maintenance (Gregg and Hedayati 2018). Accordingly, research into the pathophysiology and causes of CKD, the creation of novel, efficacious treatment approaches, and the hunt for suitable and reliable animal models of the condition are all necessary. In order to predict renal functional result and responsiveness to clinically utilized medications, the model should attempt to mimic real kidney disease in its natural course, including its histological aspects (Yang et al. 2010). One such model is adenine-induced CKD in rats and mice, which has lately seen a rise in use by a number of researchers (Ali et al. 2018, Patel et al. 2023) following its presentation by Yokozawa et al. (Yokozawa et al. 1986).

Obesity, hypertension, and diabetes mellitus are some of the factors that affect the onset and course of this CKD. In addition to these variables, oxidative stress and inflammation have been shown to play a pathophysiological role in CKD and its consequences (Ali et al. 2013). Inflammasomes, particularly NOD-like receptor protein 3 (NLRP3) inflammasome, play a significant role in the onset and development of renal disorders. NLRP3 inflammasome is activated in two stages: the initiation phase and the activation phase. Nuclear factor kappa-B (NF-κB) is activated by tumor necrosis factor (TNF) or Toll-like receptors (TLRs) during the initiation phase, which results in an up-regulation of the proteins NLRP3 and IL-1β. In the activation phase, a variety of damage-associated molecular patterns (DAMPs) cause the NLRP3 inflammasome to assemble and then become activated (Huang et al. 2023).

A non-purine selective xanthine oxidase (XO) inhibitor, febuxostat is used to treat hyperuricemia in gout patients (Yu 2007). According to Saban-Ruiz et al. (2013), it suppresses oxidative stress and lipid peroxidation (Sabán-Ruiz et al. 2013). Numerous studies have demonstrated its ability to protect the kidneys in nephrectomy rats (Sánchez-Lozada et al. 2008), as well as to inhibit apoptosis and protect against renal ischemia-reperfusion (Tsuda et al. 2012). Also, it prevents renal interstitial inflammation along with fibrosis in a model of unilateral ureteral obstructive nephropathy (Omori et al. 2012), besides ameliorating doxorubicin-induced nephrotoxicity in rats (Khames et al. 2017).

Since oxidative stress and inflammation are key components of the pathophysiology of both experimental and clinical CKD (Ali et al. 2018), we used histopathological and immunohistochemical techniques along with several established biochemical and molecular markers to test febuxostat at two graded doses against adenine induced CKD in rats.

## Materials and methods

### Animals

Thirty-two adult male Sprague-Dawley (SD) rats, (200–220 g in weight, 6–8 weeks) were acquired from “The Egyptian Organization for Biological Products and Vaccines (VACSERA)”, Giza, Egypt. During the experiment, rats were reserved at a temperature of 25 °C with a 12 h on/off light schedule. Handling of animals was in accordance with rules of Committee of Ethics of Scientific Research, Faculty of Pharmacy, Mansoura University, Egypt **(MU-ACUC (PHARM.R.23.05.20)**. That is agreed with the principles of Laboratory Animal Care (NIH publication no. 85 − 23, revised 1985).

### Drugs and chemicals

- **Febuxostat**: it was obtained as Feburic^®^ tablet from **Hikma**,** Cairo**,** Egypt**.
- **Adenine**: it was obtained as white powder from **Qualikems Fine Chemicals Pvt. Ltd. (New Delhi**,** India)**.
- The normal pellet diet was of analytical grades.

### Experimental protocol

Rats were alienated into 4 groups at random (8 rats/ group) as follows:

**Control group**: received 0.5% CMC orally for 4 weeks, **Adenine group**: received adenine (200 mg/kg/day, orally) (El-Batsh et al. 2021) suspended in 0.5% CMC for 4 weeks, **Febuxostat 5 group**: rats received adenine (200 mg/kg/day, orally) concomitantly with febuxostat (5 mg/kg/day, orally) (Xu et al. 2021) for 4 weeks and **Febuxostat 15 group**: received adenine (200 mg/kg/day, orally) concomitantly with febuxostat (15 mg/kg/ day, orally) (Amer et al. 2021) for 4 weeks.

### Determination of urinary creatinine and total protein

On day 28, each animal was immediately housed separately in metabolic cages.

for 24 h to collect urine. Samples of urine underwent centrifugation for 15 min at 3000 rpm, kept at -80 °C for further measurements.

### Sample collection

At the end of the experiment, rats were anesthetized using thiopental sodium (40 mg/kg, i.p.). The retro-orbital venous plexus was used to draw blood samples which were then centrifuged at 4000 rpm for 15 min to obtain serum. Serum samples were stored at -80 °C for further biochemical assessments. Both kidneys were collected and weighed. The left kidneys were fixed in 10% buffered formalin for histopathological and immunohistochemical analysis. The right kidneys were gathered and then divided into 2 parts; one was stored in phosphate buffered saline (PBS) for preparation of tissue homogenates and kept in -80 °C for further determination of oxidative stress biomarkers as well as inflammatory mediators. The other part was used for western blot analysis.

### Determination of kidney function biomarkers

Serum levels of creatinine (#MD1001111, Spinreact, Spain) as well as urea (#TK41041, Spinreact, Spain) were assessed via commercial assay kits as instructed by manufactures. BUN was calculated according to the following formula: BUN (mg/dl) = urea/2.14.

In addition, commercial assay kits were used for determination of creatinine (#MD1001111, Spinreact, Spain) and total protein (#MG282001, MG science and technology center, USA) in urine. Serum and urine creatinine levels were used for calculation of creatinine clearance using the following equation: CCr (ml/min) = [urine creatinine (mg/dl) × urine volume (ml) x 1440/serum creatinine (mg/dl)]. 1440: total number of minutes per day.

### Biochemical assessment

Kidney tissue homogenates were used for assessment of reduced glutathione (GSH) level and malondialdehyde (MDA) content using commercially available kits (#GR 25 10 and # MD 25 28, Biodiagnostic Co., Egypt, respectively), following the provided instructions.

Furthermore, rat ELISA kits were used to assess renal Caspase-1 (#E4594-100, biovision, USA), IL-1β (#K4796-100, biovision, USA), NLRP3 (#KD0290, avivasysbio, USA), and TNF-α (# 438204, Biolegend, USA) according to the instructions of the manufacturer.

### Western blot analysis of renal nuclear factor kappa-B (NF-κB) expression

Approximately 100 mg of the kidney was homogenized in 1 mL of TriFast reagent (Peqlab, VWR, Denmark) to extract total protein. Protein concentration was then determined using the Bradford assay method. Equal quantities of protein (30 µg per lane) were separated on SDS-PAGE, with electrophoresis performed initially at 75 V and then increased to 125 V for a total running period of about 2 h. Following separation, proteins were transferred onto a Hybond™ nylon membrane (GE Healthcare) using a TE62 Standard Transfer Tank equipped with a cooling chamber (Hoefer Inc.) and blotting buffer containing 25 mM Tris, 0.15 M NaCl, and 0.1% Tween-20 (pH 7.4) (Bradford 1976). To prevent nonspecific antibody attachment, the membrane was blocked with 5% skim milk prepared in TBST buffer (4 mM Tris base, 100 mM NaCl, 0.05% Tween-20, pH 7.5) for 1 h at room temperature. The blots were then incubated overnight at 4 °C with gentle agitation using the primary antibody of NF-κB (#PA5-16545, Invitrogen, USA, 65 kDa, 1:100). After primary antibody incubation, the membranes were washed repeatedly at room temperature for approximately 60 min using five fresh changes of wash buffer. The blots were then exposed to an HRP-conjugated anti-rabbit IgG secondary antibody (#HAF008, R&D Systems, 1:1000) for 1 h at room temperature. Protein bands were visualized using WesternBright™ ECL detection reagent (Advansta, K-12045) and captured with the Azure 600 Imaging System (Azure Biosystems). Densitometric analysis of band intensity was performed using a gel documentation unit (Geldoc-it, UVP, UK) with Totallab software (version 1.0.1). Band intensities were normalized to β-actin (#ab8227, Abcam, UK, 43 kDa, 1:1000) to control for protein loading and transfer efficiency. All Western blot analyses were conducted on 3 independent biological replicates per experimental group to ensure reproducibility.

### Histopathological and immunohistochemical assessment

Left kidneys were sectioned after being fixed in 10% formalin and embedded in paraffin wax and then stained with hematoxylin-eosin (H&E) and Masson’s trichrome (MT) stain to detect presence of fibrosis. Light microscopy was used for the analysis (Leica Imaging Systems, Cambridge, UK); the study treatment assignment was concealed from the pathologist conducting the histological evaluation.

TLR4 (#GB12186, Servicebio Biotechnology Co., China, 1:300-1:2000 dilution) was assessed via immunohistochemical technique in renal sections. In summary, deparaffinization and rehydration were followed by antigen retrieval. Rat polyclonal antibody (Thermo Scientific, Rockford, IL, USA) was used to incubate sections for an entire night at 4 °C. Following washing, the samples were visualized using diaminobenzidine after being incubated for 2 h at room temperature with goat anti-rat secondary antibody (Genemed Biotechnologies, USA). After that, the sections were examined using a Leica light microscope. Appropriate procedures were carried out to rule out background or non-specific binding (Masuda et al. 1996).

### Statistical analysis

The data are shown as mean ± standard error of mean (SE). Statistical analysis was done using GraphPad Prism software (V7, San Diego, CA, USA). Differences were deemed significant at *P* ≤ 0.05. Normality of data distribution was assessed using the Shapiro–Wilk test, while homogeneity of variance was evaluated using Levene’s test. The results obtained confirmed that the assumptions required for parametric analysis were satisfied. Therefore, one-way ANOVA followed by Tukey-Kramer’s post hoc test was applied for statistical comparisons.

## Results

### Impact of febuxostat on body weight

**Fig. 1:**
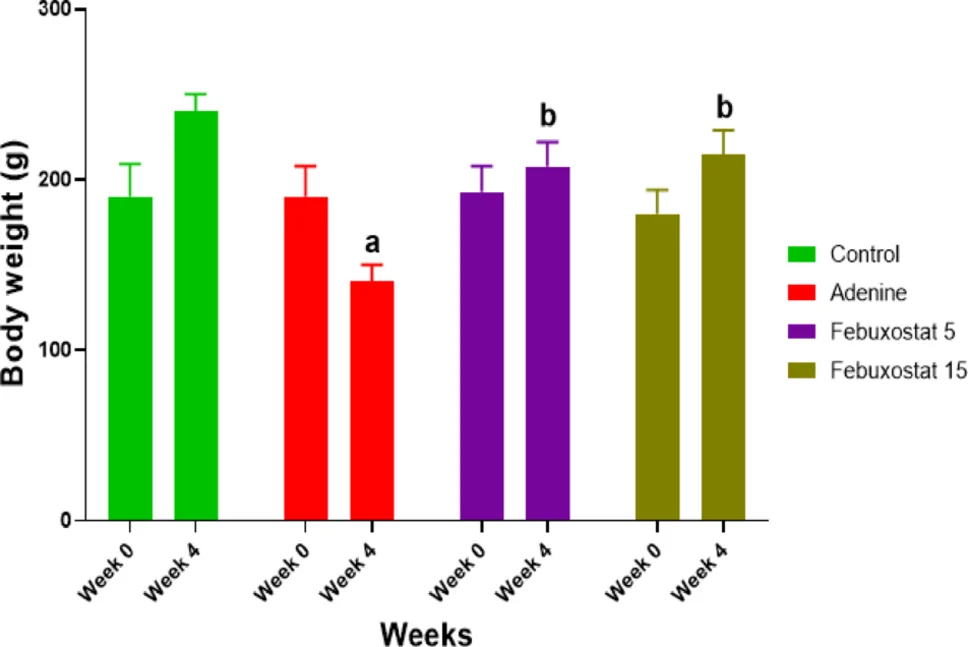
Impact of febuxostat on body weight

Administration of adenine produced a marked decrease in body by 41% relative to control group. Febuxostat administration (5 and 15 mg/kg) exhibited significant 1.5-fold increase in body weight compared to adenine group (Fig. 1).

Data are expressed as mean ± SE. Data were analyzed via ANOVA followed by Tukey-Kramer’s test. ^a^ *p* < 0.05 vs. Control, ^b^ *p* < 0.05 vs. Adenine, and ^c^ *p* < 0.05 vs. Febuxostat 5.

### Impact of febuxostat on kidney function biomarkers

In comparison to control group, adenine administration showed significant 5.8-, 8.2- and 2.4-fold increase in serum Cr, BUN and proteinuria, respectively, while revealed a significant decline in CCr.

In a dose-dependent manner, febuxostat administration (5 and 15 mg/kg) demonstrated a prompt decline in serum Cr (by 57% and 72%), BUN (by 59% and 80%) and proteinuria (by 27% and 57%) compared to adenine group. Conversely, administration of febuxostat (5 and 15 mg/kg) revealed a substantial recovery toward normal control values regarding CCr in a dose-dependent manner (Table 1).

**Table 1:** Impact of febuxostat on kidney function biomarkers

Experimental group	Serum creatinine (mg/dl)	Blood urea nitrogen (mg/dl)	Proteinuria (mg/day)	Creatinine clearance (ml/min)
Control	0.550 ± 0.009	17.42 ± 0.36	74.67 ± 3.90	1.0600 ± 0.0320
Adenine	2.920 ± 0.216 ^a^	140.68 ± 7.71 ^a^	173.17 ± 19.69 ^a^	0.0070 ± 0.0004 ^a^
Febuxostat 5	1.267 ± 0.124 ^ab^	57.55 ± 8.45 ^ab^	126.67 ± 10.33 ^ab^	0.0800 ± 0.0001 ^ab^
Febuxostat 15	0.832 ± 0.036 ^abc^	27.46 ± 1.10 ^abc^	74.33 ± 4.51 ^bc^	0.2100 ± 0.0100 ^abc^

Data are expressed as mean ± SE. Data were statistically analyzed using ANOVA followed by Tukey-Kramer’s test. ^a^ *p* < 0.05 vs. Control, ^b^ *p* < 0.05 vs. Adenine, and ^c^ *p* < 0.05 vs. Febuxostat 5

### Impact of febuxostat on oxidative/anti-oxidant balance

In comparison to control group, adenine group demonstrated a significant 5.4-fold increase in renal MDA content, while induced a significant decline in renal GSH concentration by 77%. In a dose-dependent manner, treatment with febuxostat (5 and 15 mg/kg) substantially decreased renal MDA content (by 24% and 54%, respectively) while promptly increased renal GSH level (by 2.6- and 3.2-fold, respectively) relative to adenine group (Table 2).

**Table 2:** Impact of febuxostat on oxidative/anti-oxidant balance

Experimental group	MDA (nmol/g.tissue)	GSH (mmol/g.tissue)
Control	14.270 ± 0.090	1.850 ± 0.030
Adenine	77.200 ± 4.230 ^a^	0.420 ± 0.010 ^a^
Febuxostat 5	58.480 ± 1.950 ^ab^	1.080 ± 0.010 ^ab^
Febuxostat 15	35.440 ± 2.220 ^abc^	1.350 ± 0.040 ^abc^

Data are expressed as mean ± SE. Data were statistically analyzed using ANOVA followed by Tukey-Kramer’s test. ^a^ *p* < 0.05 vs. Control, ^b^ *p* < 0.05 vs. Adenine, and ^c^ *p* < 0.05 vs. Febuxostat 5

### Impact of febuxostat on renal expression of NLRP3, Caspase-1 and NF-κB

**Fig. 2:**
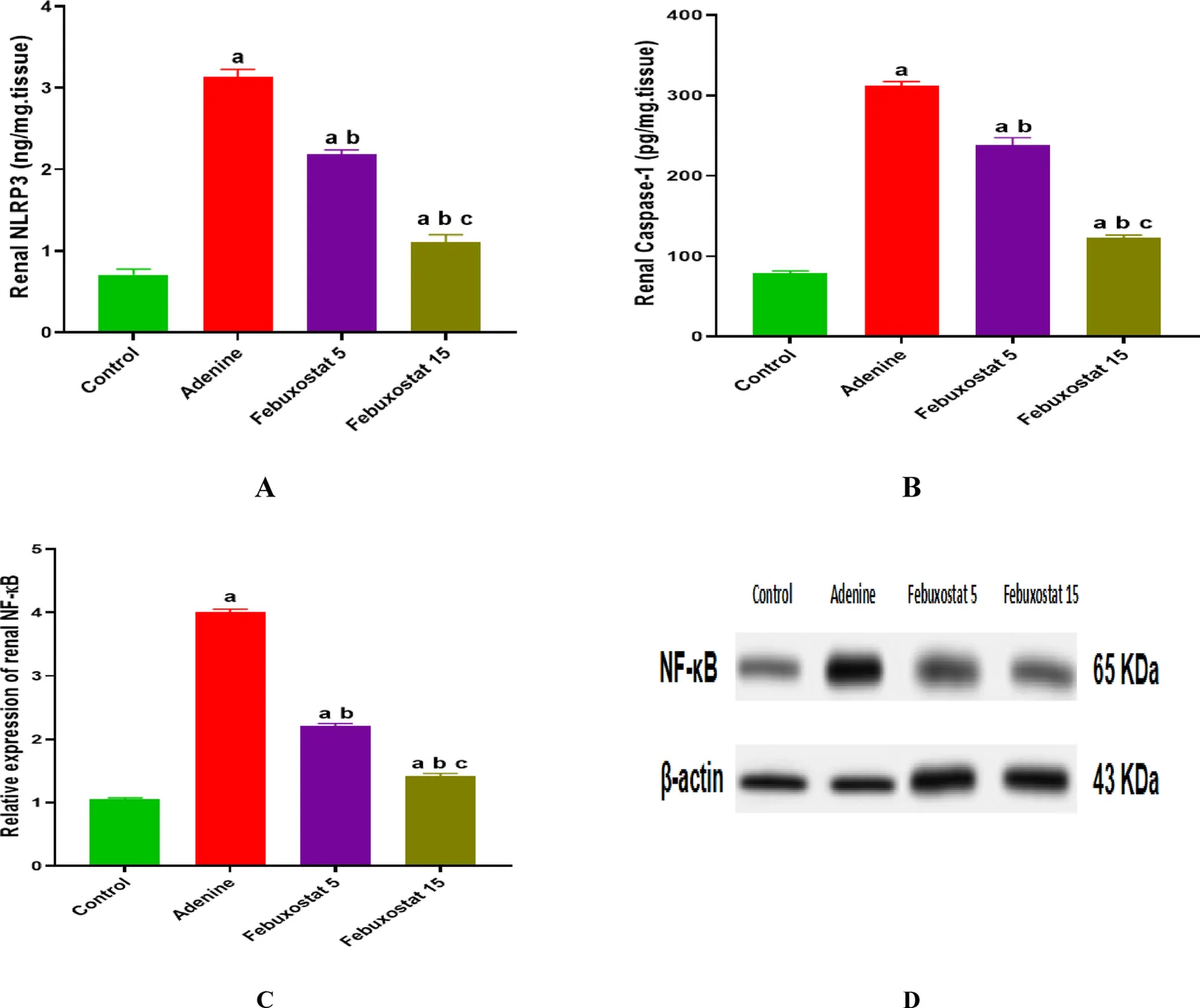
Impact of febuxostat on renal expression of NLRP3, Caspase-1 and NF-κB

As shown in Fig. 2, adenine group demonstrated a significant 4.4-, 4-and 3.8-fold increase in renal expression of NLRP3, Caspase-1 and NF-κB, respectively compared to control group. In comparison with adenine group, treatment with febuxostat (5 and 15 mg/kg) markedly reduced renal expression of NLRP3 (by 30% and 65%, respectively), Caspase-1 (by 24% and 60%, respectively) and NF-κB (by 50% and 65%, respectively) in a dose-dependent manner.

Data are expressed as mean ± SE. Data were statistically analyzed using ANOVA followed by Tukey-Kramer’s test. ^a^ *p* < 0.05 vs. Control, ^b^ *p* < 0.05 vs. Adenine, and ^c^ *p* < 0.05 vs. Febuxostat 5.

### Impact of febuxostat on renal expression of IL-1β and TNF-α

**Fig. 3:**
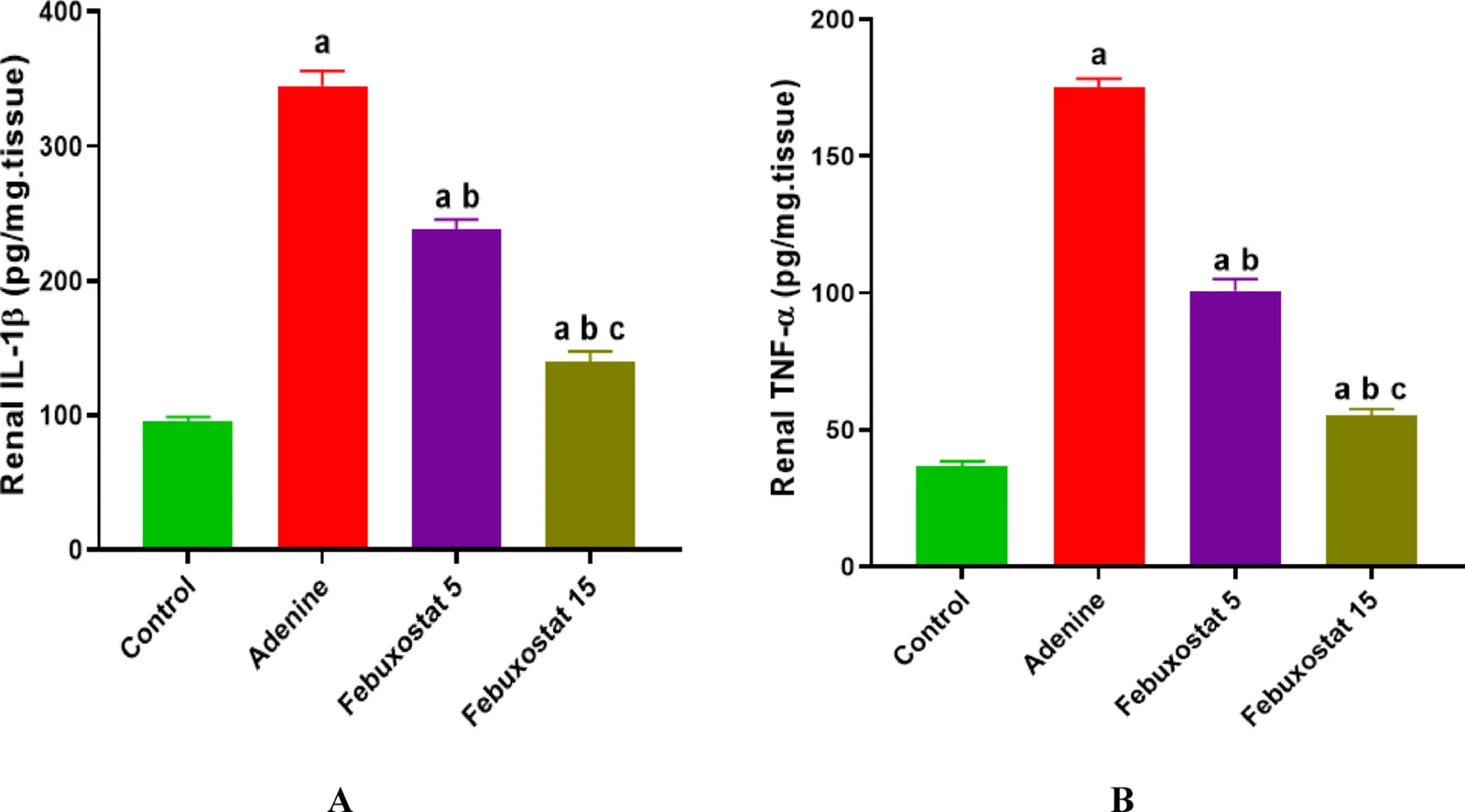
Impact of febuxostat on renal expression of IL-1β and TNF-α

In comparison to control group, adenine group revealed a significant 6- and 4.8-fold increase in renal expression of IL-1β and TNF-α, respectively. In a dose-dependent manner, administration of febuxostat (5 and 15 mg/kg) revealed a marked decline in renal expression of IL-1β (by 31% and 59%, respectively) and TNF-α (by 42% and 68%, respectively) relative to adenine group (Fig. 3).

Data are expressed as mean ± SE. Data were statistically analyzed using ANOVA followed by Tukey-Kramer’s test. ^a^ *p* < 0.05 vs. Control, ^b^ *p* < 0.05 vs. Adenine, and ^c^ *p* < 0.05 vs. Febuxostat 5.

### Impact of febuxostat on renal histopathology

Figure 4A showed normal architecture and histology of renal tissues from control rats. Whereas specimen isolated from adenine group showed adenine crystals in the proximal tubules, infiltration of inflammatory cells, dilated renal tubules, and intratubular blockage due to cellular debris and denuded epithelium, and shrunken or splitting glomeruli as evidenced by widened Bowman’s capsule (Fig. 4B). In a dose-dependent manner, treatment with febuxostat (5 and 15 mg/kg) revealed a marked improvement in adenine-induced histopathological changes in kidney specimens (Fig. 4C and D).

Masson’s Trichrome stain was used to assess renal fibrosis. As shown in Fig. 4E and F, specimen from adenine group revealed a significant 5.7-fold increase in collagen deposition relative to control group. Administration of febuxostat (5 and 15 mg/kg) demonstrated a marked reduction in collagen deposition by 58% and 77%, respectively, in comparison with adenine group in a dose-dependent manner (Fig. 4G and H). Percentage of collagen expressions in the kidney is illustrated in Fig. 4I.

Fig. 4Impact of febuxostat on renal histopathology. Renal specimen stained with H&E from **A** control group, **B** adenine group, **C**, **D** febuxostat (5 and 15 mg/kg/day). Renal specimen-stained Masson’s Trichrome stain from **E** control group, **F** adenine group, **G**, **H** febuxostat (5 and 15 mg/kg/day) and **I** effects of febuxostat (5 and 15 mg/kg/day) on collagen expression in the kidney

**Figure:**
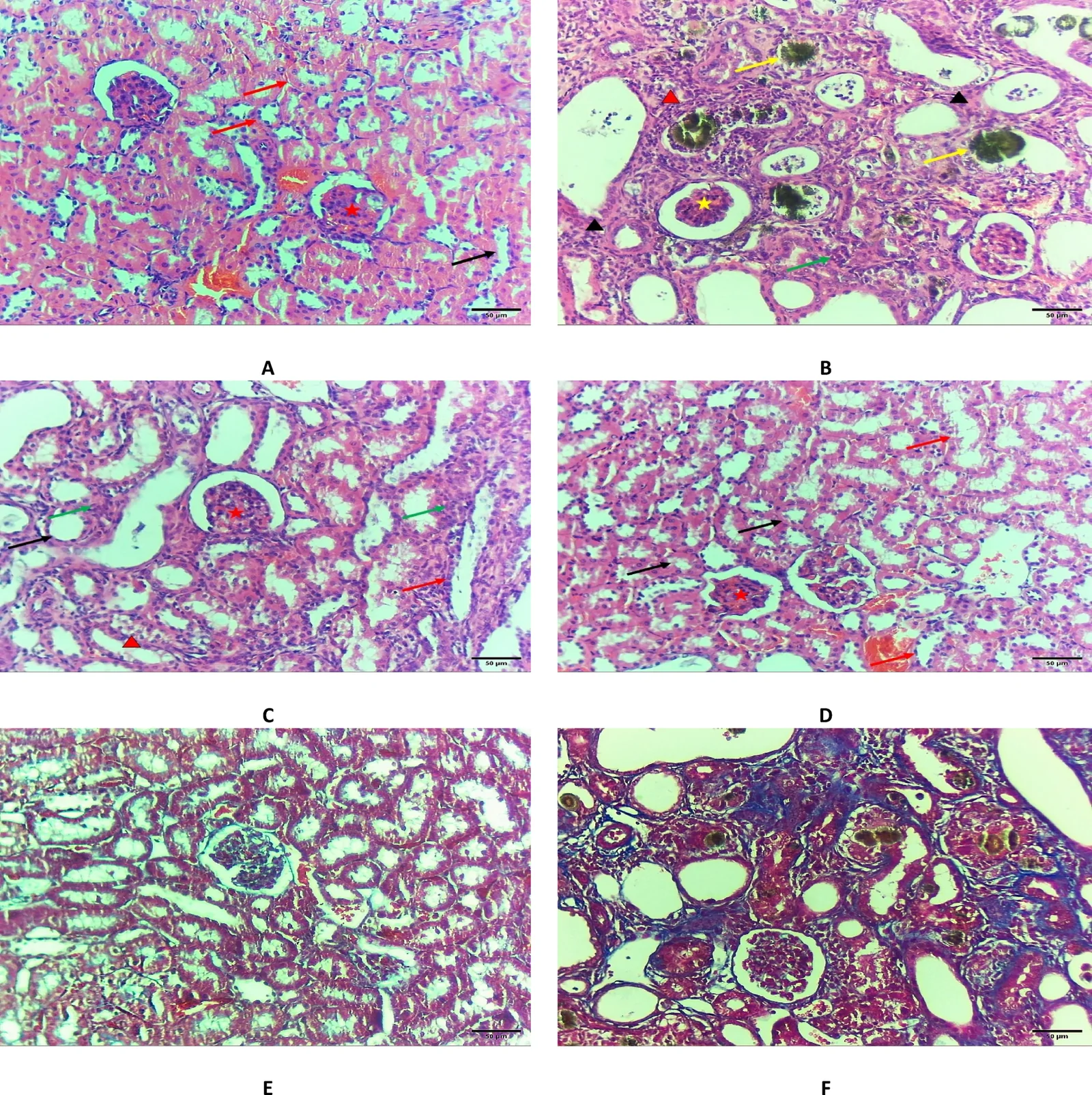

**Figure:**
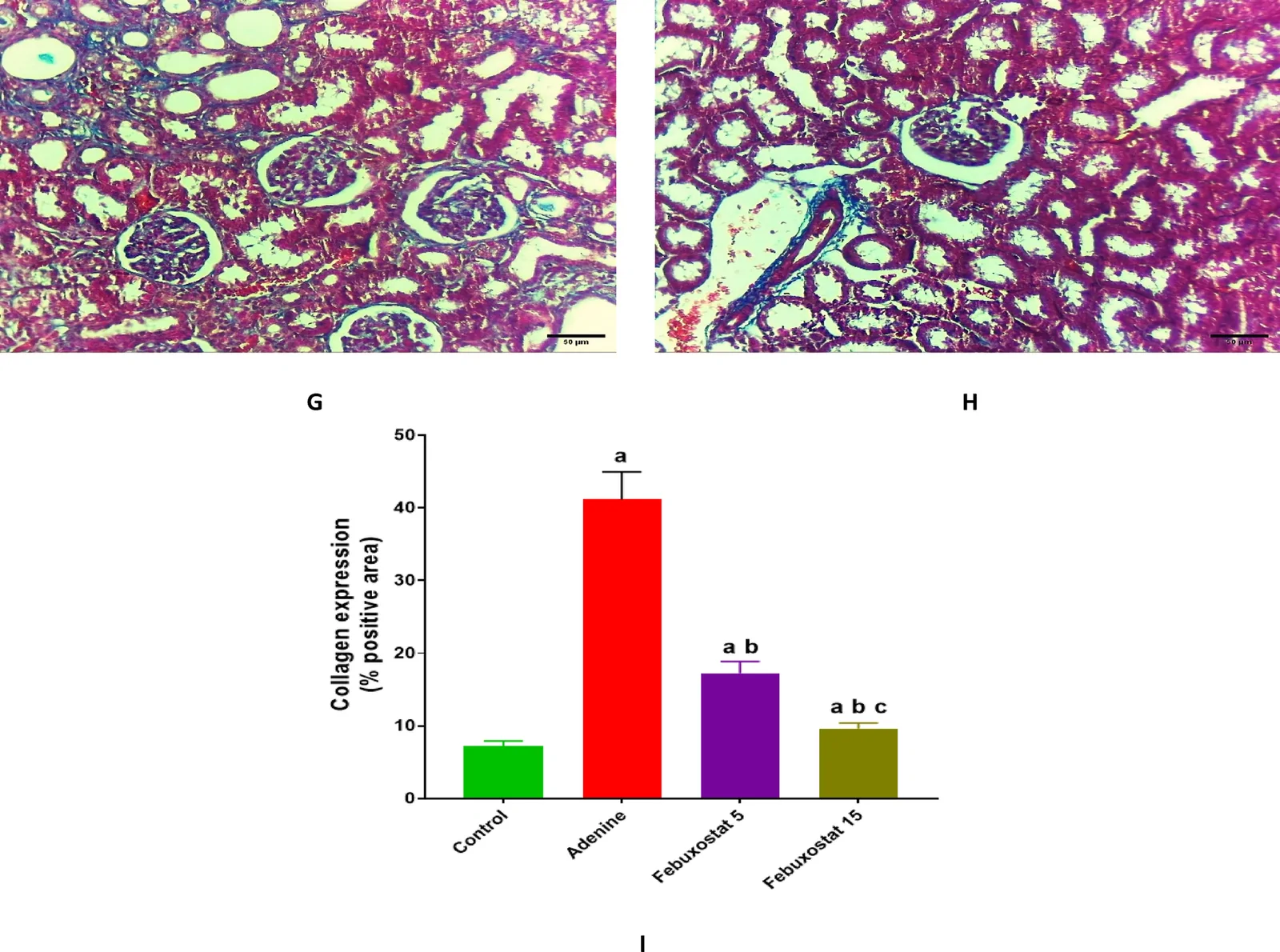


**Arrow indication:**

**Black arrow**: proximal convoluted tubules, **red arrow**: distal convoluted tubules, **red star**: normal glomeruli, **yellow arrow**: crystals of adenine in proximal tubules, **green arrow**: inflammatory cells infiltration, **black triangle**: dilated renal tubules, **red triangle**: intratubular obstruction due to the denuded epithelium and cellular debris, **yellow star**: shrunken or splitting glomeruli as evident by widened Bowman’s capsule (Magnification 200X, scale bar 50 μm).

Data are expressed as mean ± SE. Data were analyzed using ANOVA followed by Tukey-Kramer’s test. ^a^ *p* < 0.05 vs. Control, ^b^ *p* < 0.05 vs. Adenine, and ^c^ *p* < 0.05 vs. Febuxostat 5.

### Impact of febuxostat on renal TLR4 expression

**Fig. 5:**
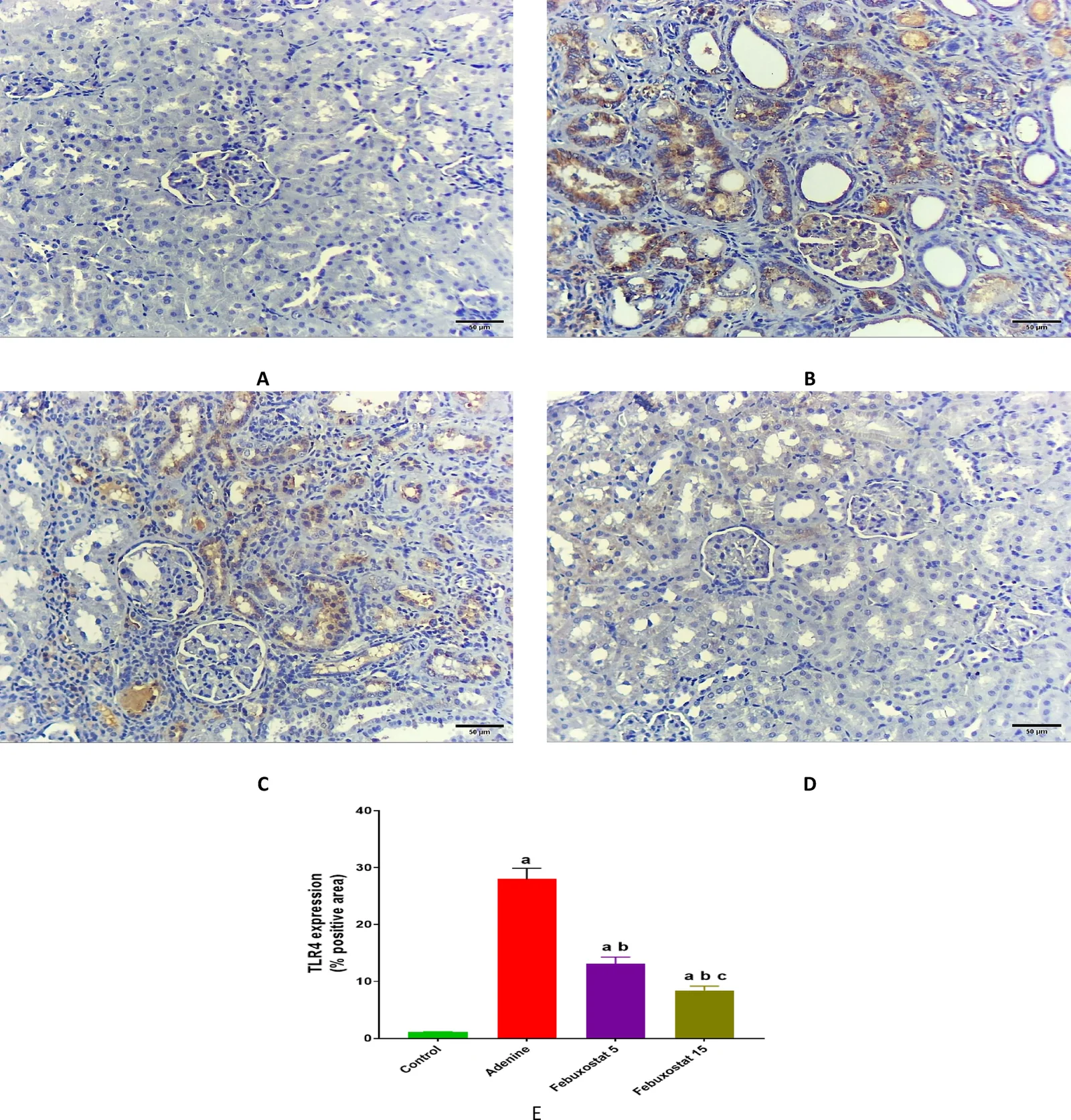
Impact of febuxostat on renal TLR4 expression. Renal specimen stained with anti-TLR4 antibody from: **A** control group, **B** adenine group **C**, **D** febuxostat (5 and 15 mg/kg/day) **E** effects of febuxostat (5 and 15 mg/kg/day) on renal TLR4 expression (Magnification 200X, scale bar 50 μm), Data are expressed as mean ± SE. Data were statistically analyzed using ANOVA followed by Tukey-Kramer’s test. ^a^ *p* < 0.05 vs. Control, ^b^ *p* < 0.05 vs. Adenine, and ^c^ *p* < 0.05 vs. Febuxostat 5.

Immunohistochemical analysis revealed that renal expression of TLR4 in adenine group was significantly increased by about 25.6-fold when compared to the normal group. Administration of febuxostat (5 and 15 mg/kg) significantly decreased renal expression of TLR4 by 53% and 70%, respectively, compared to adenine group (Fig. 5).

## Discussion

Induction of CKD in rats by adenine is a well-known model that simulates functional and structural alterations which take place in human (Samaha et al. 2023). This study examined the possible renoprotective impact of febuxostat against adenine-induced CKD. Our results revealed that oral administration of adenine for 4 weeks induced a state of CKD which was evidenced by both biochemical and histopathological (H&E staining and Masson Trichrome staining) analyses. Rats from adenine group showed marked reduction in their body weights relative to the control group; this observation could be attributed to polyuria and dehydration (Abdelrahman et al. 2019). In contrast, febuxostat in both doses effectively restored rats’ body weights.

To estimate the degree of renal impairment; serum creatinine, BUN, proteinuria and creatinine clearance were measured. Adenine significantly increased serum creatinine, BUN and proteinuria concomitant with substantial reduction in creatinine clearance which indicates impaired glomerular filtration. These findings agreed with earlier studies which reported that adenine affects the kidney function (Ali et al. 2019, Samaha et al. 2023, Abdelrahman et al. 2019). Febuxostat reduced adenine-induced CKD in the current investigation, as manifested by reduction in serum creatinine, BUN and proteinuria besides elevation in creatinine clearance. These observations came in line with prior studies which showed the renoprotective effects of febuxostat in different models such as doxorubicin-induced nephrotoxicity (Khames et al. 2017) and hyperuricemic nephropathy (He et al. 2017).

These biochemical findings were further confirmed by histopathological examination which revealed that adenine group showed infiltration of inflammatory cells, dilated renal tubules, intratubular blockage caused by denuded epithelium and cellular debris, and shrinking or splitting glomeruli indicated by an enlarged Bowman’s capsule besides crystals of in proximal tubules. These crystals may represent 2,8dihydroxyadenine, a poorly soluble metabolite of adenine in rats that form crystals in the tubules, resulting in kidney damage (Diwan et al. 2018). Contrarily, febuxostat in both doses resulted in improvement in histopathological alterations induced by adenine confirming its renoprotective effect.

It has been reported that oxidative stress as well as inflammation are major contributors in the development of CKD (Ali et al. 2019). Correspondingly, our data revealed that oral administration of adenine for 4 weeks resulted in disturbed oxidant/antioxidant balance in addition to triggered inflammatory state. This was demonstrated by a notable increase in MDA content and a notable decrease in GSH level relative to the control group, supporting the results of earlier studies which stated that adenine induced oxidative stress (Ali et al. 2018, Ali et al. 2013). On the other hand, treatment with febuxostat is associated with the reduction of MDA in the kidney, which has been raised by adenine, and the recovery of GSH, the main antioxidant defense molecule, and thus implied its potential as an antioxidant. This is consistent with previous research which showed that febuxostat reduced oxidative stress induced by doxorubicin (Khames et al. 2017). XO enzyme produces reactive oxygen species (ROS) and encourages lipid peroxidation (Ardan et al. 2004). Thus, we can deduce that febuxostat can combat oxidative stress because it is a strong XO inhibitor.

Many endogenous ligands are produced by immune cells or injured tubular cells during chronic tubulointerstitium injury. These ligands have the potential to over-activate TLR4 expression which phosphorylates and activates NF-κB (Lepenies et al. 2011). Phosphorylated NF-κB translocates into the nucleus upon activation, then becomes attached to certain inflammatory gene sites to initiate transcription and then produce a significant quantity of proinflammatory cytokines; IL-1β and TNF-α (Sakr et al. 2025, Balaha et al. 2023). In adenine-induced CKD, it has been documented that activation of TLR4 signaling pathway contributed to the inflammation of the tubulointerstitium (Chen et al. 2016). In line with the earlier investigation, this study revealed increased expression of TLR4 and NF-κB as well as renal levels of IL-1β and TNF-α in rats with adenine-induced CKD relative to the normal rats. Conversely, treatment with febuxostat showed substantial reduction in expression of TLR4 and NF-κB as well as renal levels of IL-1β and TNF-α compared to adenine group. These findings are consistent with a prior study which reported that febuxostat protected against acetaminophen-induced liver inflammation via inhibiting TLR4/NF-κB pathway (Tian and Zhang 2023).

To further focus on the mechanistic pathway by which febuxostat exerts its renoprotective effect, NLRP3/Caspase1/IL-1β pathway was assessed. As previously mentioned, TLR4 activation stimulates NLRP3 activation, which in turn triggers apoptosis by upregulating ASC and activating apoptotic biomarkers (Zaafar et al. 2022). Also, oxidative stress, caused by increase in ROS and other free radicals within cells, is linked to NLRP3 inflammasome activation (Huang et al. 2023). In the current investigation, NLRP3 and Caspase-1 activation coexisted with the TLR-4 activation found in tissue samples from adenine-treated rats. These elevated levels were involved in the activation of oxidative stress and proinflammatory pathways. These findings agreed with former research which reported increased expression of NLRP3 in adenine-fed rats (Tian et al. 2023). Alongside the nephroprotection that febuxostat provided, these indicators were inhibited. These results were consistent with a previous study that demonstrated that febuxostat exerted its nephroprotective impact by inhibiting TLR4, NLRP3, ASC and Caspase-1 (Abdel-Wahab et al. 2023).

**Fig. 6:**
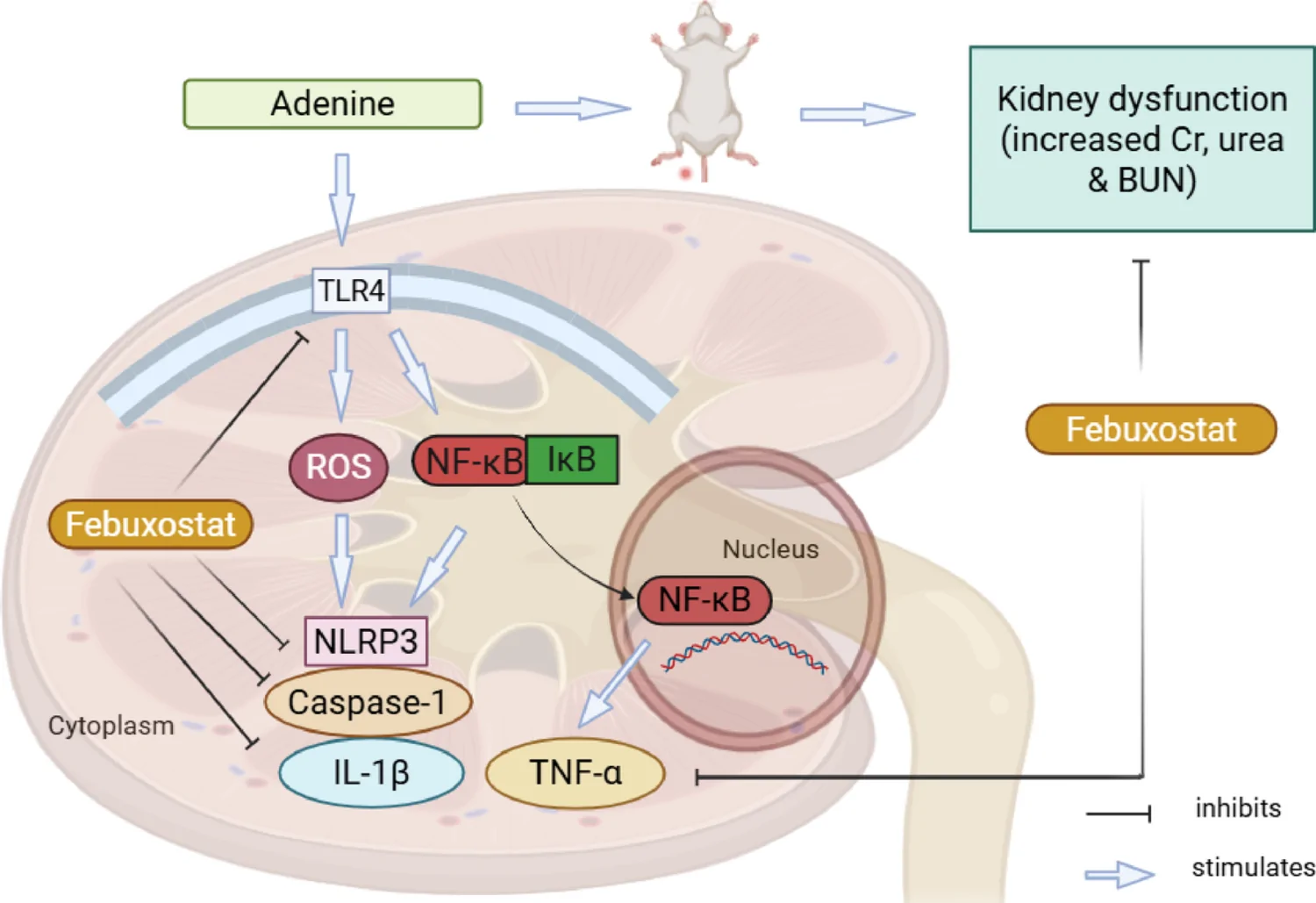
Schematic presentation illustrating the mechanistic pathway of febuxostat against adenine-induced CKD

In conclusion, febuxostat could have renoprotective impact in the context of adenine-induced CKD via inhibiting TLR4/NF-κB/NLRP3/Caspase-1/IL-1β signaling pathway (Fig. 6). Clinical research is required to validate our hypotheses and certify these findings.

## Study limitations

A limitation of the current study is the absence of direct functional validation. Specifically, genetic knockdown or specific chemical inhibitors to definitively confirm the pathway. Moreover, the absence of a febuxostat-treated normal control group, which limits the ability to completely exclude any independent effects of febuxostat on normal renal tissue under physiological conditions. Furthermore, this model does not fully recapitulate CKD in general, but rather predominantly reflects tubulointerstitial fibrosis and tubular injury.

## Data Availability

Data will be made available on request.
